# Insights into the Link between the Organization of DNA Replication and the Mutational Landscape

**DOI:** 10.3390/genes10040252

**Published:** 2019-03-27

**Authors:** Julia Gaboriaud, Pei-Yun Jenny Wu

**Affiliations:** CNRS, University of Rennes, Institute of Genetics and Development of Rennes, 35043 Rennes, France; julia.gaboriaud@univ-rennes1.fr

**Keywords:** DNA replication, replication program, mutational landscape, genome dynamics, genome instability

## Abstract

The generation of a complete and accurate copy of the genetic material during each cell cycle is integral to cell growth and proliferation. However, genetic diversity is essential for adaptation and evolution, and the process of DNA replication is a fundamental source of mutations. Genome alterations do not accumulate randomly, with variations in the types and frequencies of mutations that arise in different genomic regions. Intriguingly, recent studies revealed a striking link between the mutational landscape of a genome and the spatial and temporal organization of DNA replication, referred to as the replication program. In our review, we discuss how this program may contribute to shaping the profile and spectrum of genetic alterations, with implications for genome dynamics and organismal evolution in natural and pathological contexts.

## 1. Introduction

The faithful duplication and transmission of the genetic material is critical for cell proliferation as well as for development and differentiation. Indeed, errors in DNA synthesis may give rise to mutations that are deleterious for an organism, leading to disease or death. The duplication of the genetic material is tightly regulated to promote genome integrity, giving rise to a spatial and temporal pattern, or program of DNA replication. Interestingly, the replication program has been shown to be strongly correlated with the genetic variation found in eukaryotic genomes. In this review, we present findings from a variety of organisms that link the replication program with mutation frequency, distribution, and spectra. We also discuss the mechanisms by which the replication program may be involved in shaping the accumulation of mutations across a genome. Importantly, the conservation of the coupling between the organization of DNA replication and the mutational landscape from yeast to humans suggests a key role for the replication program in genome evolution.

## 2. The Spatial and Temporal Organization of DNA Replication

DNA replication is an essential step of the cell cycle that is highly controlled to ensure that the genetic material is entirely replicated once and only once prior to cell division. DNA synthesis begins at sites called origins of replication, and bi-directional extension from these sites ultimately produces a complete copy of the genome. In contrast to bacteria, where the genome is duplicated by well-defined origins that fire once per cell cycle [1], DNA replication in eukaryotes displays a more complex organization. A large number of replication origins are distributed throughout eukaryotic genomes, ranging from hundreds in budding yeast to tens of thousands in human cells [2]. Not all origins are fired during each synthesis (S) phase, and different subsets are activated from one cell cycle to the next [3]. The activity of an origin is characterized by two major parameters: its timing of firing during S phase, and its efficiency, or frequency of usage in a population of cells. The timings and efficiencies of origin usage, together with the distribution of origins along the chromosomes, define the program of DNA replication.

**Figure 1:**
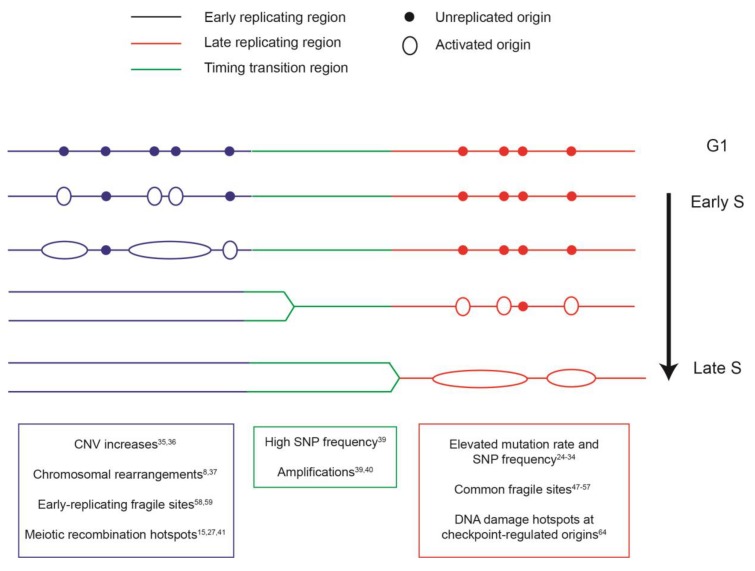
Schematic of the replication program and genome instability events associated with different replication domains. Colors correspond to the timing of replication of a giving region in the synthesis (S) phase. Blue: early-replicating domain, red: late-replicating domain, and green: timing transition region. Unreplicated origins are indicated by closed circles; initiated and elongating origins are shown as open circles and ovals. The distinct genome instability features that are enriched in each replication domain are indicated in the boxes below, with the relevant references noted. CNV: copy number variation. SNP: single-nucleotide polymorphism.

A large body of work has demonstrated that eukaryotic DNA replication is a temporally and spatially regulated process. DNA synthesis is organized in chromosomal domains that are copied at distinct times during S phase [4,5,6,7,8] (Figure 1). In addition, genomic regions that are duplicated at the same time are localized near one another. Replication domain boundaries coincide with those of chromatin compartments known as topologically associating domains (TADs), indicating a close coupling between the replication program and large-scale chromosome structure [9]. Furthermore, the replication program is linked to the three-dimensional arrangement of the genome in the nucleus. Pulse-labeling of cells with nucleoside analogs revealed discrete sites of DNA synthesis called replication foci that co-localize with components of the replication machinery [10]. This spatial pattern of DNA replication changes during S phase, as early-replicating regions are located inside the nucleus, while late domains are found near the nuclear periphery [11,12]. Altogether, these findings demonstrate a multi-scale organization of DNA replication in eukaryotes.

Interestingly, replication programs are strongly conserved between related species [8,13], suggesting evolutionary constraints for these architectures. However, the organization of DNA replication is flexible and responds to environmental and developmental cues [14,15,16,17], and recent studies suggested that the replication program may make critical contributions to cellular physiology. Replication timing is associated with chromatin state, as early duplication is correlated with actively transcribed euchromatin, while heterochromatic regions are generally late-replicating [16,18]. Related to these observations, alterations in replication patterns during development and differentiation are accompanied by changes in transcriptional activity and epigenetic marks [17,19,20,21,22]. In addition, a significant level of cell-type specific conservation of replication timing profiles was observed between mouse and human cells [7]. Importantly, experimental evidence points to direct roles for the replication program in regulating histone gene transcription in budding yeast [23] and meiotic recombination in fission yeast [15]. Thus, the accumulating links between gene expression, chromatin structure, and the organization of DNA replication indicate that this feature may be a key modulator of cellular function.

Recently, the replication program was also suggested to be involved in the acquisition of genetic diversity. Mutations serve as substrates for selection and evolution, and it has long been known that they do not accumulate randomly across a genome. A remarkable correlation between replication timing, mutation frequency, and mutation spectrum has emerged from work on a variety of organisms [24,25,26,27]. These connections indicate that the replication program may be a crucial input that affects the types and distributions of genetic alterations that arise in different genomic regions. In the following sections, we highlight these associations and discuss how the organization of DNA replication may contribute to the genetic variation that is central to evolution.

## 3. Coupling between the Replication Program and Mutational Landscape

During the process of DNA replication, the genetic material is susceptible to being damaged and acquiring mutations. Cells therefore possess mechanisms to limit these challenges to genome integrity. However, genome instability and errors in DNA synthesis are important sources of the genetic alterations that are necessary for evolution. While previous studies demonstrated that mutation rate and distribution are non-uniform across the genome, we still do not understand how this variation is generated. Recent studies have established an interplay between replication timing and the mutational landscape in diverse systems, and we present some of these findings below.

First, late-replicating regions are associated with higher mutation rates. In the budding yeast *Saccharomyces cerevisiae*, assessment of mutation frequency across chromosome VI using a genetic assay revealed a six-fold variation that is correlated with replication timing, with earlier regions displaying lower mutation rates [25]. These results are consistent with studies of single-nucleotide polymorphisms (SNPs) identified between 39 strains of *S. cerevisiae*, which showed that mutation rate in a region increases as replication occurs later in S phase [28]. Similarly, analysis of genome-wide replication timing data and polymorphisms in the fruit fly *Drosophila melanogaster* uncovered a 30% increase in mutation rate between the latest and earliest replicating sequences [29]. Along the same lines, work in mammalian systems provided evidence for a correspondence between the replication timing of genomic regions and their associated mutation rates. Indeed, comparisons of evolutionary divergence and nucleotide diversity in human and mouse genome sequencing datasets [24,30], as well as of different human cell lines [26,27], indicate that mutation rate is significantly increased in late-replicating regions. This is also the case in cancer cells, where mutation frequencies are two- to three-fold higher in late- vs. early-replicating areas [31,32,33,34]. Collectively, these findings bring to light a coupling between replication timing and mutation rate in both normal and pathological contexts.

Second, the types of mutations that accumulate across a genome are correlated with replication timing. For instance, in cancer cells, copy number variation (CNV) increases are more frequently found in early-replicating regions, whereas deletions are enriched in late-replicating domains [35]. This relationship was likewise observed during the reprogramming of human induced pluripotent stem cells, as CNV increases accumulate in genomic regions that become early-replicating during this process [36]. In addition, early-replicating domains are more likely to harbor large-scale rearrangements, such as for those that differentiate mouse and human genomes [8] or for chromosomal translocations in hematological cancer cells, which lead to gene fusions that drive cancer progression [37]. Moreover, analysis of human genomes revealed that structural mutations mediated by homology-based recombination mechanisms were enriched in regions that are copied early in S phase [26]. These studies therefore suggest that replication timing is associated not only with the frequency but also the types of genetic alterations that arise in different genomic regions.

While the findings described above focus on early vs. late replicating regions, areas located between such domains also have a characteristic mutation phenotype. These timing transition regions (TTR) are characterized by a progressive change in replication timing, and they contain few or no replication origins. TTRs are often duplicated by replication fork progression from nearby initiation sites, leading to a higher probability of fork stalling [38]. This feature may be part of what gives rise to the genome instability and elevated SNP frequency that are found in these regions [39]. Interestingly, analysis of TTRs in human chromosomes 11q and 21q revealed that they contain amplification events and translocations associated with cancer, as well as synteny breakpoints between the mouse and human genomes [39,40]. Thus, although it is not clear how the mutational phenotype of TTRs relates to those described above for early- and late-replicating domains, these transition areas may be a distinctive source of genetic variation.

The replication program may also participate in generating genome diversity during sexual reproduction. Meiotic recombination provides genetic variation, and hotspots of recombination have been identified in numerous organisms. Comparison of the mouse meiotic recombination landscape with replication profiles indicated that early-replicating regions harbor a higher density of such hotspots [27]. A similar correlation was observed in human genomes in a study of crossover recombination in parent–child pairs [41]. This relationship is supported by experimental evidence in fission yeast, where changes in the replication program were demonstrated to induce corresponding alterations in the distribution of meiotic double-stranded DNA break (DSB) formation that is central to recombination [15]. Indeed, for a given genomic region, increasing origin efficiencies resulted in increases in meiotic DSB formation and recombination frequencies. These findings suggest a role for the organization of DNA replication in modulating the profile of genetic variation during meiosis.

Taken together, the studies described above establish a compelling link between the organization of DNA replication, the rate of mutation, and the spectrum of genome alterations in eukaryotic genomes (Figure 1).

## 4. The Replication Program and Genome Instability Hotspots

In addition to the correlation between the organization of DNA replication and the genome-wide mutation landscape, the replication program is associated with instability at specific genomic loci in both normal and challenging conditions. For example, a key genomic feature whose duplication must be coordinated with cell-cycle progression is the centromere, which is crucial for mediating chromosome segregation during mitosis and meiosis. Centromeric structure differs among eukaryotes, ranging from extended heterochromatic regions in most organisms to point centromeres without heterochromatin in budding yeast. Nevertheless, centromeres are replicated in early S phase in fungi and in at least a subset of more complex eukaryotes [42,43,44,45]. Despite this conservation, the importance of this specific timing remained an open question. In budding yeast, the early duplication of the centromere was suggested to aid in preserving genome integrity. In the context of replication stress conditions and a checkpoint mutant in which centromeres are not duplicated, Feng and colleagues showed that the chromosome segregation defect in this background is dependent on the timing of centromere replication [46]. These results indicate that early centromere duplication during a critical time window may promote the establishment of bioriented chromosomes for proper segregation and cell division in the budding yeast. However, given the differences in centromeric structure between budding yeast and other eukaryotes, further studies will be required to generalize these conclusions.

Next, genome instability occurs at loci called fragile sites that were identified in the genomes of eukaryotes ranging from yeast to humans [47,48]. Common fragile sites (CFSs) preferentially form gaps or breaks in metaphase chromosomes in conditions where replication is challenged. Most of the known CFSs can be induced by aphidicolin, an inhibitor of DNA polymerase [47,49]. These sites are hotspots of genome instability, and they participate in sister chromatid exchange, deletions, translocations, and gene amplifications [48,50,51,52]. Moreover, they are recognized as sites of DNA damage and chromosomal rearrangement in different cancers [53,54]. One hallmark of CFSs is their late replication during S phase. This is clearly the case for *FRA3B*, one of the earliest identified fragile sites and the most frequently observed CFS in human lymphocytes [47]. Not only is this locus late-replicating, but treatment with aphidoicolin further delays its duplication [55]. *FRA3B* was found to be depleted of replication initiation events, and it is flanked by origins that fire in mid-S phase; this is also seen at *FRA16D*, the second most common CFS in lymphocytes [56]. Notably, these features are linked to the instability of both *FRA3B* and *FRA16D*, as these loci are not fragile in cell types that do not display this replication initiation and timing profile. Along the same lines, a recent study showed that induced early replication of a CFS is accompanied by a reduction in its fragility [57]. Altogether, these findings implicate replication timing as a key regulator of the landscape of CFS instability.

Although the majority of fragile sites are associated with late-replicating regions, a subset of early-replicating fragile sites (ERFSs) has been identified [58]. Analysis of the profile of DNA damage in murine B cells treated with hydroxyurea to generate replication stress uncovered replication fork collapse in early-replicating genomic regions. In contrast to CFSs, ERFSs are located near replication initiation sites. They are found in regions with a higher gene density, and their fragility is increased by transcriptional activity. Similarly, induction of the oncogenes *CCNE1* (cyclin E1) and *MYC* in a human cell line leads to ectopic firing of origins located within highly transcribed genes [59]. Although such events are normally inhibited by transcription through these origins during gap 1 (G1) phase, oncogene overexpression brings about early S phase entry before completion of transcription at these loci, leading to unscheduled firing at these sites. The subsequent conflicts between replication and transcription result in replication fork collapse, formation of double-stranded DNA breaks, and chromosomal rearrangements. Thus, collisions between the replication and transcription machineries may play a role in the instability of early-replicating fragile sites.

Intriguingly, the sites of replication initiation themselves may also be involved in genome plasticity. Studies of genome architecture, experimental evolution, and DNA repair all have associated replication origins with genetic variability [50,60,61,62]. For instance, comparative analyses of genome rearrangements and gene amplifications found in budding yeast species revealed that these alterations are often bounded by origins [50,62]. Similarly, early-firing origins were correlated with breakpoints between *S. cerevisiae* and *Lachancea waltii* [50], two yeasts that are diverged by ~150 million years. Such a relationship was likewise uncovered in evolved vs. ancestral strains from laboratory evolution experiments, where the presence of origins at rearrangement sites before breakage suggests that they may participate in these events [50]. Complementary to these findings, increased mutation rates are associated with origins of replication in budding yeast. Using mutation accumulation assays to analyze spontaneous mutations that arise in the absence of selective pressure, Lujan et al. found a higher rate of indels near the autonomously replicating sequence (ARS) consequence sequence (ACS) motifs in replication origins [63]. Furthermore, in fission yeast cells exposed to replication stress, origins in late-replicating regions that are normally inhibited by the checkpoint become hotspots of DNA damage when they are fired inappropriately [64]. These results, therefore, indicate that replication origins may make unique contributions to genetic diversity.

## 5. Mechanisms Underlying the Profile of Genetic Variation

Although the studies described above provide evidence for a close coupling between the replication program and the genome-wide mutational landscape, we are only beginning to understand the mechanisms that are responsible for this interplay. The variation in genetic alterations that arises along the chromosomes is due to a combination of the processes that generate genome instability and errors in DNA synthesis, as well as those that deal with these problems.

A number of mechanisms were proposed to account for the increased mutation rate that is associated with late S phase. One major source of genome instability is the slowing and stalling of replication forks. This leads to generation of single-stranded DNA (ssDNA), which is more prone to damage, breakage, and mutation than double-stranded DNA [65,66,67]. Replication fork progression is challenged by a variety of endogenous stresses, including an insufficient level of factors that are required for DNA synthesis. First, a balanced supply of deoxyribonucleotide triphosphates (dNTPs) is critical for genome integrity, with a maximal concentration observed during S phase [68]. Replication fork velocity is sensitive to small changes in dNTP level [69,70], and reductions or mild imbalances among the individual dNTPs are mutagenic [71]. Rates of replication errors due to abnormally elevated deoxycytidine triphosphate (dCTP) and deoxythymidine triphosphate (dTTP) concentrations were found to be elevated in late-replicating regions [72], which may suggest a greater sensitivity to dNTP levels as these building blocks are consumed during S phase. Second, during the process of DNA synthesis, replication protein A (RPA) binds to ssDNA and protects stalled replication forks. Exposure to replication stress of human cells inhibited for ataxia telangiectasia and Rad3-related protein (ATR) checkpoint function leads to an excess of ssDNA that exhausts the available pool of RPA [73], resulting in double-stranded DNA breaks. Although this global RPA exhaustion was shown to occur during a perturbed S phase in sensitized conditions, it is possible that RPA may become limiting in certain growth conditions or genomic regions during normal cell proliferation. Third, accurate duplication of the genome requires the associated copying of its chromatin landscape. This is disrupted by the passage of replication forks and must be restored on the daughter DNA strands. Histone production is cell-cycle regulated, and reducing histone supply slows DNA synthesis during S phase [74,75,76]. Importantly, sufficient levels of histone proteins are required to maintain genome integrity. Replication fork velocity is linked to histone synthesis and to assembly of newly synthesized DNA into nucleosomes [77], and decreased histone H4 expression in budding yeast leads to impaired replication fork progression and increased homologous recombination [78]. Collectively, the observations described above indicate that a limiting supply of key factors required for DNA and chromatin replication may be partly responsible for a higher mutation rate during late S phase.

In addition, natural impediments to DNA replication in the genome can promote replication fork stalling and collapse. For example, tight DNA–protein associations and chromatin compaction render heterochromatin more difficult to replicate, and specific chromatin remodeling complexes are required to promote replication through such regions [79]. Indeed, the euchromatin vs. heterochromatin organization of the genome is suggested to be a major determinant of mutation rate variation along the chromosomes. Analysis of cancer genomes revealed that increased mutation rates are strongly correlated with closed chromatin, in particular with the heterochromatin-associated H3K9me3 histone modification [33,80]. Another crucial obstacle for replication forks involves DNA-bound transcription complexes, with collisions between replication and transcription machineries resulting in genome instability [81,82]. Head-on encounters between these processes are more mutagenic than co-directional conflicts, leading to replication fork pausing and an increase in recombination [83,84]. Complementary to these findings, genes that are highly transcribed by RNA polymerase II were identified as barriers for the replication machinery in budding yeast [85]. Furthermore, concomitant replication and transcription on the same template is linked to the instability of late-replicating CFSs in human cells [86]. Interestingly, deleterious encounters appear to have been minimized through evolution, such as through favoring co-directional replication and transcription, as well as their spatial and temporal organization [87,88,89,90]. However, this is not sufficient to avoid conflicts between these two processes; for instance, CFSs are often located in very long genes (>800 kb) whose transcription takes more than one cell cycle, and delaying replication does not allow for the separation of these two processes [86]. These findings, therefore, demonstrate that interactions between chromatin structure, transcription, and replication are critical contributors to genome instability.

Finally, the pathways via which cells manage DNA damage and errors also represent key sources of the differences in mutation rate and spectrum that arise across a genome. Upon encountering DNA lesions that block normal DNA polymerases, cells can use two processes to replicate past these sites: template switching, which is non-mutagenic, or translesion synthesis (TLS), which has a high error rate [91]. TLS polymerases are not expressed until late S phase [92,93], and they are not available to repair lesions that arise in early S phase. In budding yeast, disruption of TLS results in a reduction in the mutation frequency of a late-replicating region but has no significant effect on early-replicating sites [25]. In addition, analysis of primate divergence data indicates that the mutation signature for the TLS polymerase ζ is more frequently found in late- vs. early-replicating regions [94]. The timing of replication of late regions may then make them more susceptible to be repaired by error-prone TLS polymerases, consequently increasing their mutation rates. Furthermore, recent evidence implicates DNA mismatch repair (MMR) as a crucial contributor to elevated mutation rates in late-replicating regions. MMR corrects base–base and insertion–deletion mismatches, and it was shown to be less effective in late S phase [63]. Importantly, a recent study of single-nucleotide variants from cancer genomes provides compelling evidence that MMR generates regional variations in mutation frequency [95]. The authors observed that MMR-deficient tumors exhibited an equalization of the distribution of mutations along the chromosomes: losing MMR earlier during tumor progression was linked to lower differences in regional mutation rates. This suggests that genetic alterations that arise in tumors after MMR inactivation are not enriched in late-replicating regions, thus abrogating the coupling between replication timing and mutation frequency. Altogether, these studies identify differential DNA repair as a major factor in generating regional variations in mutation rate.

## 6. Conclusions

Mutations are fundamental to the biology of living organisms. They are an essential source of genetic diversity for evolution and play a critical role in disease. Although it was documented early on that mutation rates vary across a genome, the mechanisms that determine the landscape of genetic alterations remain poorly understood. Recently, the organization of DNA replication has been strongly correlated with the distribution and types of mutations that accumulate throughout a genome. Early-replicating regions of the genome are enriched for large-scale rearrangements, translocations, CNV increases, and meiotic recombination hotspots, while late-replicating areas have higher mutation rates, elevated SNP levels, and CFSs. As many of these associations were revealed through analyses of sequencing data, a causal role for the replication program in establishing the genome-wide profile of genetic variation remains to be evaluated. Moreover, the mechanisms via which the replication program contributes to this profile remain to be elucidated, and future studies will determine the processes that are responsible for how the replication program may be coupled to different frequencies and types of genetic alterations in a genome, in both normal and pathological contexts.

Although the essential function of DNA replication is to produce an accurate copy of the genetic material, accumulating evidence suggests the intriguing possibility that the replication program may be a crucial contributor to genetic diversity. Understanding this novel aspect of the organization of DNA replication will have important implications for our knowledge of the processes that drive the adaptation and evolution of living organisms.

## References

[1] Mott M.L., Berger J.M. (2007). DNA replication initiation: Mechanisms and regulation in bacteria. Nat. Rev. Microbiol..

[2] Méchali M. (2010). Eukaryotic DNA replication origins: Many choices for appropriate answers. Nat. Rev. Mol. Cell Biol..

[3] Patel P.K., Arcangioli B., Baker S.P., Bensimon A., Rhind N. (2006). DNA replication origins fire stochastically in fission yeast. Mol. Biol. Cell.

[4] Taljanidisz J., Popowski J., Sarkar N. (1989). Temporal order of gene replication in Chinese hamster ovary cells. Mol. Cell. Biol..

[5] Desprat R., Thierry-Mieg D., Lailler N., Lajugie J., Schildkraut C., Thierry-Mieg J., Bouhassira E.E. (2009). Predictable dynamic program of timing of DNA replication in human cells. Genome Res..

[6] Heichinger C., Penkett C.J., Bähler J., Nurse P. (2006). Genome-wide characterization of fission yeast DNA replication origins. EMBO J..

[7] Ryba T., Hiratani I., Lu J., Itoh M., Kulik M., Zhang J., Schulz T.C., Robins A.J., Dalton S., Gilbert D.M. (2010). Evolutionarily conserved replication timing profiles predict long-range chromatin interactions and distinguish closely related cell types. Genome Res..

[8] Yaffe E., Farkash-Amar S., Polten A., Yakhini Z., Tanay A., Simon I. (2010). Comparative analysis of DNA replication timing reveals conserved large-scale chromosomal architecture. PLoS Genet..

[9] Pope B.D., Ryba T., Dileep V., Yue F., Wu W., Denas O., Vera D.L., Wang Y., Hansen R.S., Canfield T.K. (2014). Topologically associating domains are stable units of replication-timing regulation. Nature.

[10] Malyavantham K.S., Bhattacharya S., Alonso W.D., Acharya R., Berezney R. (2008). Spatio-temporal dynamics of replication and transcription sites in the mammalian cell nucleus. Chromosoma.

[11] Berezney R., Dubey D.D., Huberman J.A. (2000). Heterogeneity of eukaryotic replicons, replicon clusters, and replication foci. Chromosoma.

[12] Heun P., Laroche T., Shimada K., Furrer P., Gasser S.M. (2001). Chromosome dynamics in the yeast interphase nucleus. Science.

[13] Muller C.A., Nieduszynski C.A. (2012). Conservation of replication timing reveals global and local regulation of replication origin activity. Genome Res..

[14] Perrot A., Millington C.L., Gómez-Escoda B., Schausi-Tiffoche D., Wu P.-Y.J. (2018). CDK activity provides temporal and quantitative cues for organizing genome duplication. PLoS Genet..

[15] Wu P.-Y.J., Nurse P. (2014). Replication origin selection regulates the distribution of meiotic recombination. Mol. Cell.

[16] Pope B.D., Hiratani I., Gilbert D.M. (2010). Domain-wide regulation of DNA replication timing during mammalian development. Chromosome Res..

[17] Hiratani I., Ryba T., Itoh M., Yokochi T., Schwaiger M., Chang C.-W., Lyou Y., Townes T.M., Schübeler D., Gilbert D.M. (2008). Global reorganization of replication domains during embryonic stem cell differentiation. PLoS Biol..

[18] Stambrook P.J., Flickinger R.A. (1970). Changes in chromosomal DNA replication patterns in developing frog embryos. J. Exp. Zool..

[19] Rodríguez-Martínez M., Pinzón N., Ghommidh C., Beyne E., Seitz H., Cayrou C., Méchali M. (2017). The gastrula transition reorganizes replication-origin selection in Caenorhabditis elegans. Nat. Struct. Mol. Biol..

[20] Siefert J.C., Georgescu C., Wren J.D., Koren A., Sansam C.L. (2017). DNA replication timing during development anticipates transcriptional programs and parallels enhancer activation. Genome Res..

[21] MacAlpine D.M., Rodríguez H.K., Bell S.P. (2004). Coordination of replication and transcription along a Drosophila chromosome. Genes Dev..

[22] Pourkarimi E., Bellush J.M., Whitehouse I. (2016). Spatiotemporal coupling and decoupling of gene transcription with DNA replication origins during embryogenesis in *C. elegans*. eLife.

[23] Müller C.A., Nieduszynski C.A. (2017). DNA replication timing influences gene expression level. J. Cell Biol..

[24] Stamatoyannopoulos J.A., Adzhubei I., Thurman R.E., Kryukov G.V., Mirkin S.M., Sunyaev S.R. (2009). Human mutation rate associated with DNA replication timing. Nat. Genet..

[25] Lang G.I., Murray A.W. (2011). Mutation rates across budding yeast chromosome VI are correlated with replication timing. Genome Biol. Evol..

[26] Koren A., Polak P., Nemesh J., Michaelson J.J., Sebat J., Sunyaev S.R., McCarroll S.A. (2012). Differential relationship of DNA replication timing to different forms of human mutation and variation. Am. J. Hum. Genet..

[27] Yehuda Y., Blumenfeld B., Mayorek N., Makedonski K., Vardi O., Cohen-Daniel L., Mansour Y., Baror-Sebban S., Masika H., Farago M. (2018). Germline DNA replication timing shapes mammalian genome composition. Nucleic Acids Res..

[28] Agier N., Fischer G. (2012). The mutational profile of the yeast genome is shaped by replication. Mol. Biol. Evol..

[29] Weber C.C., Pink C.J., Hurst L.D. (2012). Late-replicating domains have higher divergence and diversity in Drosophila melanogaster. Mol. Biol. Evol..

[30] Chen C.-L., Rappailles A., Duquenne L., Huvet M., Guilbaud G., Farinelli L., Audit B., d’Aubenton-Carafa Y., Arneodo A., Hyrien O. (2010). Impact of replication timing on non-CpG and CpG substitution rates in mammalian genomes. Genome Res..

[31] Woo Y.H., Li W.-H. (2012). DNA replication timing and selection shape the landscape of nucleotide variation in cancer genomes. Nat. Commun..

[32] Liu L., De S., Michor F. (2013). DNA replication timing and higher-order nuclear organization determine single-nucleotide substitution patterns in cancer genomes. Nat. Commun..

[33] Polak P., Karlić R., Koren A., Thurman R., Sandstrom R., Lawrence M., Reynolds A., Rynes E., Vlahoviček K., Stamatoyannopoulos J.A. (2015). Cell-of-origin chromatin organization shapes the mutational landscape of cancer. Nature.

[34] Lawrence M.S., Stojanov P., Polak P., Kryukov G.V., Cibulskis K., Sivachenko A., Carter S.L., Stewart C., Mermel C.H., Roberts S.A. (2013). Mutational heterogeneity in cancer and the search for new cancer-associated genes. Nature.

[35] De S., Michor F. (2011). DNA replication timing and long-range DNA interactions predict mutational landscapes of cancer genomes. Nat. Biotechnol..

[36] Lu J., Li H., Hu M., Sasaki T., Baccei A., Gilbert D.M., Liu J.S., Collins J.J., Lerou P.H. (2014). The distribution of genomic variations in human iPSCs is related to replication-timing reorganization during reprogramming. Cell Rep..

[37] Shugay M., Ortiz de Mendíbil I., Vizmanos J.L., Novo F.J. (2012). Genomic hallmarks of genes involved in chromosomal translocations in hematological cancer. PLoS Comput. Biol..

[38] Guan Z., Hughes C.M., Kosiyatrakul S., Norio P., Sen R., Fiering S., Allis C.D., Bouhassira E.E., Schildkraut C.L. (2009). Decreased replication origin activity in temporal transition regions. J. Cell Biol..

[39] Watanabe Y., Fujiyama A., Ichiba Y., Hattori M., Yada T., Sakaki Y., Ikemura T. (2002). Chromosome-wide assessment of replication timing for human chromosomes 11q and 21q: Disease-related genes in timing-switch regions. Hum. Mol. Genet..

[40] Watanabe Y., Ikemura T., Sugimura H. (2004). Amplicons on human chromosome 11q are located in the early/late-switch regions of replication timing. Genomics.

[41] Halldorsson B.V., Palsson G., Stefansson O.A., Jonsson H., Hardarson M.T., Eggertsson H.P., Gunnarsson B., Oddsson A., Halldorsson G.H., Zink F. (2019). Characterizing mutagenic effects of recombination through a sequence-level genetic map. Science.

[42] McCarroll R.M., Fangman W.L. (1988). Time of replication of yeast centromeres and telomeres. Cell.

[43] Ahmad K., Henikoff S. (2001). Centromeres are specialized replication domains in heterochromatin. J. Cell Biol..

[44] Kim S.-M., Dubey D.D., Huberman J.A. (2003). Early-replicating heterochromatin. Genes Dev..

[45] Koren A., Tsai H.-J., Tirosh I., Burrack L.S., Barkai N., Berman J. (2010). Epigenetically-inherited centromere and neocentromere DNA replicates earliest in S-phase. PLoS Genet.

[46] Feng W., Bachant J., Collingwood D., Raghuraman M.K., Brewer B.J. (2009). Centromere replication timing determines different forms of genomic instability in Saccharomyces cerevisiae checkpoint mutants during replication stress. Genetics.

[47] Glover T.W., Berger C., Coyle J., Echo B. (1984). DNA polymerase α inhibition by aphidicolin induces gaps and breaks at common fragile sites in human chromosomes. Hum. Genet..

[48] Admire A., Shanks L., Danzl N., Wang M., Weier U., Stevens W., Hunt E., Weinert T. (2006). Cycles of chromosome instability are associated with a fragile site and are increased by defects in DNA replication and checkpoint controls in yeast. Genes Dev..

[49] Glover T.W., Wilson T.E., Arlt M.F. (2017). Fragile sites in cancer: More than meets the eye. Nat. Rev. Cancer.

[50] Di Rienzi S.C., Collingwood D., Raghuraman M.K., Brewer B.J. (2009). Fragile genomic sites are associated with origins of replication. Genome Biol. Evol..

[51] Debatisse M., Le Tallec B., Letessier A., Dutrillaux B., Brison O. (2012). Common fragile sites: Mechanisms of instability revisited. Trends Genet..

[52] Arlt M.F., Durkin S.G., Ragland R.L., Glover T.W. (2006). Common fragile sites as targets for chromosome rearrangements. DNA Repair.

[53] Arlt M.F., Miller D.E., Beer D.G., Glover T.W. (2002). Molecular characterization of FRAXB and comparative common fragile site instability in cancer cells. Genes Chromosomes Cancer.

[54] Burrow A.A., Williams L.E., Pierce L.C.T., Wang Y.-H. (2009). Over half of breakpoints in gene pairs involved in cancer-specific recurrent translocations are mapped to human chromosomal fragile sites. Bmc Genom..

[55] Le Beau M.M., Rassool F.V., Neilly M.E., Espinosa R., Glover T.W., Smith D.I., McKeithan T.W. (1998). Replication of a common fragile site, FRA3B, occurs late in S phase and is delayed further upon induction: Implications for the mechanism of fragile site induction. Hum. Mol. Genet..

[56] Letessier A., Millot G.A., Koundrioukoff S., Lachagès A.-M., Vogt N., Hansen R.S., Malfoy B., Brison O., Debatisse M. (2011). Cell-type-specific replication initiation programs set fragility of the FRA3B fragile site. Nature.

[57] Blin M., Le Tallec B., Nähse V., Schmidt M., Brossas C., Millot G.A., Prioleau M.-N., Debatisse M. (2019). Transcription-dependent regulation of replication dynamics modulates genome stability. Nat. Struct. Mol. Biol..

[58] Barlow J.H., Faryabi R.B., Callén E., Wong N., Malhowski A., Chen H.T., Gutierrez-Cruz G., Sun H.-W., McKinnon P., Wright G. (2013). Identification of early replicating fragile sites that contribute to genome instability. Cell.

[59] Macheret M., Halazonetis T.D. (2018). Intragenic origins due to short G1 phases underlie oncogene-induced DNA replication stress. Nature.

[60] Hwang J.-Y., Smith S., Ceschia A., Torres-Rosell J., Aragón L., Myung K. (2008). Smc5-Smc6 complex suppresses gross chromosomal rearrangements mediated by break-induced replications. DNA Repair.

[61] Dunn B., Sherlock G. (2008). Reconstruction of the genome origins and evolution of the hybrid lager yeast Saccharomyces pastorianus. Genome Res..

[62] Gordon J.L., Byrne K.P., Wolfe K.H. (2009). Additions, losses, and rearrangements on the evolutionary route from a reconstructed ancestor to the modern Saccharomyces cerevisiae genome. PLoS Genet..

[63] Lujan S.A., Clausen A.R., Clark A.B., MacAlpine H.K., MacAlpine D.M., Malc E.P., Mieczkowski P.A., Burkholder A.B., Fargo D.C., Gordenin D.A. (2014). Heterogeneous polymerase fidelity and mismatch repair bias genome variation and composition. Genome Res..

[64] Gómez-Escoda B., Wu P.-Y.J. (2018). The organization of genome duplication is a critical determinant of the landscape of genome maintenance. Genome Res..

[65] Feng W., Di Rienzi S.C., Raghuraman M.K., Brewer B.J. (2011). Replication stress-induced chromosome breakage is correlated with replication fork progression and is preceded by single-stranded DNA formation. G3.

[66] Roberts S.A., Sterling J., Thompson C., Harris S., Mav D., Shah R., Klimczak L.J., Kryukov G.V., Malc E., Mieczkowski P.A. (2012). Clustered mutations in yeast and in human cancers can arise from damaged long single-strand DNA regions. Mol. Cell.

[67] Chan K., Sterling J.F., Roberts S.A., Bhagwat A.S., Resnick M.A., Gordenin D.A. (2012). Base damage within single-strand DNA underlies in vivo hypermutability induced by a ubiquitous environmental agent. PLoS Genet..

[68] Chabes A., Georgieva B., Domkin V., Zhao X., Rothstein R., Thelander L. (2003). Survival of DNA damage in yeast directly depends on increased dNTP levels allowed by relaxed feedback inhibition of ribonucleotide reductase. Cell.

[69] Poli J., Tsaponina O., Crabbé L., Keszthelyi A., Pantesco V., Chabes A., Lengronne A., Pasero P. (2012). dNTP pools determine fork progression and origin usage under replication stress. EMBO J..

[70] Wilhelm T., Ragu S., Magdalou I., Machon C., Dardillac E., Técher H., Guitton J., Debatisse M., Lopez B.S. (2016). Slow replication fork velocity of Homologous recombination-defective cells results from Endogenous oxidative stress. PLoS Genet..

[71] Kumar D., Viberg J., Nilsson A.K., Chabes A. (2010). Highly mutagenic and severely imbalanced dNTP pools can escape detection by the S-phase checkpoint. Nucleic Acids Res..

[72] Watt D.L., Buckland R.J., Lujan S.A., Kunkel T.A., Chabes A. (2016). Genome-wide analysis of the specificity and mechanisms of replication infidelity driven by imbalanced dNTP pools. Nucleic Acids Res..

[73] Toledo L.I., Altmeyer M., Rask M.-B., Lukas C., Larsen D.H., Povlsen L.K., Bekker-Jensen S., Mailand N., Bartek J., Lukas J. (2013). ATR prohibits replication catastrophe by preventing global exhaustion of RPA. Cell.

[74] Hereford L.M., Osley M.A., Ludwig T.R., McLaughlin C.S. (1981). Cell-cycle regulation of yeast histone mRNA. Cell.

[75] Osley M.A. (1991). The regulation of histone synthesis in the cell cycle. Annu. Rev. Biochem..

[76] Zhao X., McKillop-Smith S., Müller B. (2004). The human histone gene expression regulator HBP/SLBP is required for histone and DNA synthesis, cell cycle progression and cell proliferation in mitotic cells. J. Cell Sci..

[77] Mejlvang J., Feng Y., Alabert C., Neelsen K.J., Jasencakova Z., Zhao X., Lees M., Sandelin A., Pasero P., Lopes M. (2014). New histone supply regulates replication fork speed and PCNA unloading. J. Cell Biol..

[78] Prado F., Aguilera A. (2005). Partial depletion of histone H4 increases homologous recombination-mediated genetic instability. Mol. Cell. Biol..

[79] Collins N., Poot R.A., Kukimoto I., García-Jiménez C., Dellaire G., Varga-Weisz P.D. (2002). An ACF1-ISWI chromatin-remodeling complex is required for DNA replication through heterochromatin. Nat. Genet..

[80] Schuster-Böckler B., Lehner B. (2012). Chromatin organization is a major influence on regional mutation rates in human cancer cells. Nature.

[81] French S. (1992). Consequences of replication fork movement through transcription units in vivo. Science.

[82] Deshpande A.M., Newlon C.S. (1996). DNA replication fork pause sites dependent on transcription. Science.

[83] Prado F., Aguilera A. (2005). Impairment of replication fork progression mediates RNA polII transcription-associated recombination. EMBO J..

[84] Paul S., Million-Weaver S., Chattopadhyay S., Sokurenko E., Merrikh H. (2013). Accelerated gene evolution through replication-transcription conflicts. Nature.

[85] Azvolinsky A., Giresi P.G., Lieb J.D., Zakian V.A. (2009). Highly transcribed RNA polymerase II genes are impediments to replication fork progression in Saccharomyces cerevisiae. Mol. Cell.

[86] Helmrich A., Ballarino M., Tora L. (2011). Collisions between replication and transcription complexes cause common fragile site instability at the longest human genes. Mol. Cell.

[87] Wansink D.G., Manders E.E., van der Kraan I., Aten J.A., van Driel R., de Jong L. (1994). RNA polymerase II transcription is concentrated outside replication domains throughout S-phase. J. Cell Sci..

[88] Wang J.D., Berkmen M.B., Grossman A.D. (2007). Genome-wide coorientation of replication and transcription reduces adverse effects on replication in Bacillus subtilis. Proc. Natl. Acad. Sci. USA.

[89] Huvet M., Nicolay S., Touchon M., Audit B., d’Aubenton-Carafa Y., Arneodo A., Thermes C. (2007). Human gene organization driven by the coordination of replication and transcription. Genome Res..

[90] Marsolier-Kergoat M.-C., Goldar A. (2012). DNA replication induces compositional biases in yeast. Mol. Biol. Evol..

[91] Sale J.E. (2013). Translesion DNA synthesis and mutagenesis in eukaryotes. Cold Spring Harb. Perspect. Biol..

[92] Friedberg E.C. (2005). Suffering in silence: The tolerance of DNA damage. Nat. Rev. Mol. Cell Biol..

[93] Plachta M., Halas A., McIntyre J., Sledziewska-Gojska E. (2015). The steady-state level and stability of TLS polymerase eta are cell cycle dependent in the yeast *S. cerevisiae*. DNA Repair.

[94] Seplyarskiy V.B., Bazykin G.A., Soldatov R.A. (2015). Polymerase ζ activity is linked to replication timing in Humans: evidence from mutational signatures. Mol. Biol. Evol..

[95] Supek F., Lehner B. (2015). Differential DNA mismatch repair underlies mutation rate variation across the human genome. Nature.

